# Editorial: Flattening the Curve

**DOI:** 10.1016/j.shj.2023.100215

**Published:** 2023-08-04

**Authors:** Tsuyoshi Kaneko, Connor P. Callahan

**Affiliations:** Division of Cardiothoracic Surgery, Washington University School of Medicine, St. Louis, Missouri, USA

The Ozaki procedure (aortic valve neocuspidization) was first reported by Dr. Shigeyuki Ozaki in 2011. This procedure involves the recreation of the aortic valve leaflets using autologous pericardium.^1^ The proposed benefit of this procedure was longer coaptation length and preserved annular mobility leading to a larger orifice area. Midterm results have shown durable outcomes. However, its adaptation has been slow.^2^ One of the reasons is the complex nature of the procedure, which involves autologous pericardial harvesting, resecting and creating the shape of each leaflet, and suturing to the annulus. To overcome complexity, a dedicated tool has been developed, which includes the sizers and the cusp template with a detailed guide to leaflet suturing.

In this edition of the journal, Patel and colleagues have presented a standardized approach adopted at the Cleveland Clinic to minimize the learning curve of the Ozaki procedure. Their strategy included limiting the operators to 2 surgeons, using the dedicated tools to standardize the procedure, and most importantly, visiting Dr. Ozaki to observe the surgery and installing wet lab practice. Notably, in their first 20 cases, there was no major perioperative morbidity or mortality, while the cardiopulmonary bypass and aortic cross-clamp times steadily decreased by 20 ​minutes. Both cardiopulmonary time and aortic cross-clamp time plateaued after 20 cases. These steps proved quite effective given the minimum perioperative morbidity in their early experience and relatively rapid decrease in bypass and cross-clamp times. The authors emphasize that the 2 keys to minimizing their learning curve were observation and coaching from an expert, Dr Ozaki, and simulation in the wet lab.

The authors are to be commended, as there is a learning curve for every procedure that we do, especially complex procedures. For context, other examples of challenging cardiac operations that can have a significant learning curve in the field of cardiac surgery include valve-sparing aortic root replacement and minimally invasive mitral valve repair. Beckmann and colleagues^3^ described their learning curve with valve-sparing aortic root replacement. They found that less surgeon experience was a significant risk factor for aortic valve-related reoperation-free survival. Interestingly, 20 cases were needed to start seeing a decrease in aortic cross-clamp time and aortic valve reoperation. The reduction in both was seen even after 40 cases. Other series have indicated that the learning curve continued for up to 7 years in terms of aortic valve-related reoperations.^4^ For minimally invasive mitral surgery, Vo et al.^5^ described valve repair requiring at least 90 cases to have an acceptable technical complication rate. By taking adequate strategies, the Cleveland Clinic group was able to diminish the learning curve significantly, considering the complexity of the procedure.

As highlighted in this article, simulation in the wet lab was vital to minimize their learning curve. As we advance, the simulation will be the key to improving outcomes in our field, specifically by minimizing the learning curve for every procedure we do. For example, congenital cardiac surgery is another segment of our field with a significant learning curve for junior surgeons and trainees and an area where outcomes have come under increasing scrutiny. The group at the Hospital for Sick Children in Toronto has published its Hands-On Surgical Training program. Their trainees and others at outside institutions participate monthly in three-dimensional (3D)-printed models for specific congenital cardiac lesions. This program has the benefit of hands-on training on challenging anatomy and repairs and further facilitates coaching from expert surgeons.^6^ All participants in their program have found it to help improve their surgical skills.^7^ As 3D printing becomes easier to streamline, using models both for congenital and adult cardiac procedures will make the learning curve less steep for trainees and junior surgeons when they step into the operating room. With the advancement in the field of simulation and 3D printing, it is critical to leverage these to decrease the learning curve in a complex procedure.

This study highlights that all surgeons will inevitably experience a learning curve with a new operation, even with a master surgeon. There is some concern about the early aortic regurgitation with this procedure, and adaptation to the general population needs further investigation. However, this manuscript proves that adequate preparation and steps can flatten the learning curve. Most importantly, these dogmas are also applicable to nonsurgical procedures. There are numerous upcoming technologies in the field of structural heart disease, such as transcatheter mitral/tricuspid repair/replacement. These procedures are often done by the heart team, including interventional cardiologists and/or cardiac surgeons. As we embrace the new innovations in the field of structural heart, we as a specialty need to constantly think of how to flatten the learning curve while maintaining the quality of the procedure. The authors' experience provides a roadmap for all of us to try to emulate.

## Funding

The authors have no funding to report.

## Disclosure Statement

T. Kaneko is a consultant for Medtronic, Edwards Lifesciences, and Abbott Vascular. The other author had no conflicts to declare.

## References

[1] Ozaki S., Kawase I., Yamashita H. (2014). A total of 404 cases of aortic valve reconstruction with glutaraldehyde-treated autologous pericardium. J Thorac Cardiovasc Surg.

[2] Pirola S., Mastroiacovo G., Arlati F. (2020). Single center 5-years’ experience of Ozaki procedure: mid-term follow-up. Ann Thorac Surg.

[3] Beckmann E., Martens A., Krueger H. (2020). Aortic valve-sparing root replacement (David): learning curve and impact on outcome. Interact Cardiovasc Thorac Surg.

[4] Chirichilli I., Scaffa R., Irace F.G. (2023). Twenty-year experience of aortic valve reimplantation using the Valsalva graft. Eur J Cardio Thorac Surg.

[5] Vo A.T., Nguyen D.H., Van Hoang S. (2019). Learning curve in minimally invasive mitral valve surgery: a single-center experience. J Cardiothorac Surg.

[6] Van Arsdell G.S., Hussein N., Yoo S.J. (2020). Three-dimensional printing in congenital cardiac surgery-Now and the future. J Thorac Cardiovasc Surg.

[7] Yoo S.J., Spray T., Austin E.H., Yun T.J., van Arsdell G.S. (2017). Hands-on surgical training of congenital heart surgery using 3-dimensional print models. J Thorac Cardiovasc Surg.

